# Impact of the introduction of the AMNOG law on launch delays of new drugs in Germany: a comment

**DOI:** 10.1007/s10198-023-01607-5

**Published:** 2023-06-24

**Authors:** Afschin Gandjour

**Affiliations:** Frankfurt School of Finance & Management, Adickesallee 32-34, 60322 Frankfurt am Main, Germany

**Keywords:** I11, I18

A study by Büssgen and Stargardt [1] aimed at analyzing how the enactment of the German Arzneimittelmarktneuordnungsgesetz (AMNOG), which was introduced in Germany in 2011 to mandate early benefit assessments of new, innovative drugs, impacted launch delays in Germany compared to other European countries between 2011 and 2017. Launch delay was defined “as the length of time between the first international launch date of each pharmaceutical [worldwide] and its corresponding national launch date in a given country.” Specifically, the authors compared launch delays in Germany with “those in five EU centralized and regulated countries that (a) had not had any major regulatory interventions related to HTA or reimbursement after 2011 and (b) had a similar pre-AMNOG trend from 2003 to 2009.”

To conduct the analysis, the authors extracted “[t]he first international launch date (worldwide) and national launch dates of prescription pharmaceuticals […] from the IQVIA (former IMS) MIDAS Sales Database.” The authors found that “[a] cross our full sample of 30 countries, Germany's ranking worsened slightly from a rank 3.79 in the pre-AMNOG period to a rank 3.93 in the post-AMNOG period.” Furthermore, they stated that “Germany has never been the first country to launch (0% of our sample before AMNOG and in 0% of our sample after AMNOG).” Moreover, while “the launch delay in Germany and all five comparator countries decreased significantly from the pre-AMNOG period to the post-AMNOG period, [...] the introduction of AMNOG in Germany consistently reduced the magnitude of the decrease in launch delay compared to each of the five comparator countries.”

The authors concluded that “AMNOG […] may lead to longer launch delays and thus later patient access in Germany.” Furthermore, they see a “trade-off between regulation and access to care.”

In the following, I would like to discuss and challenge the above findings and conclusions. First, the result that Germany ranks 3.93 in the post-AMNOG period is at odds with an analysis conducted by IQVIA itself. According to IQVIA [2], Germany had the shortest time to availability of new, innovative medicines between 2017 and 2020 in Europe (on average, 133 days starting from the time of marketing authorization). With regard to subsets of drugs, this holds for orphan drugs, oncological drugs, and non-oncology orphan drugs but not for combination therapies (IQVIA 2022). Therefore, it is vital that the authors reconcile the findings from their analysis of IQVIA’s database with those reported by IQVIA itself. Second, the five European countries chosen as comparator countries had longer launch delays before the enactment of AMNOG. Thus, they are not directly comparable to Germany. If there is a trend towards a reduction in delay in all countries, a catch-up effect in these countries is not surprising. Similarly, as countries move toward reducing delay, the opportunity for same-size reductions in delay becomes smaller. Therefore, it appears more plausible to compare relative reductions in delays, which adjust for differences at baseline. Based on this operationalization, Germany’s reduction of launch delay is approximately average (42% vs. 43%).

The authors’ conclusion that “AMNOG […] may lead to longer launch delays and thus later patient access in Germany” is not true given that Germany showed a reduction in launch delays in the post-AMNOG period. Even if interpreted as a “smaller reduction in launch delays,” the conclusion does not hold when adding the determiner “relative” (“smaller relative reduction in launch delays”). Furthermore, the claimed “trade-off between regulation and access to care” is not apparent given Germany’s continuing position as the first launch country in Europe (on average), at least if we trust IQVIA’s analysis.

## Data Availability

All data are publicly available.
